# Study on temperature change and nursing intervention of patients undergoing thoracoscopic surgery in lung tumor treatment

**DOI:** 10.1097/MD.0000000000040672

**Published:** 2024-12-13

**Authors:** Xiao Qu, Na Hu, Liyan Zhou

**Affiliations:** a Operating Room, Affiliated Hospital of Jiangnan University, Wuxi, Jiangsu, China; b Department of Anesthesiology, Affiliated Hospital of Jiangnan University, Wuxi, Jiangsu, China.

**Keywords:** lung tumor, nursing intervention, temperature change, thoracoscopic surgery

## Abstract

To investigate the changes of body temperature and nursing intervention of patients undergoing thoracoscopic surgery in lung tumor treatment. A total of 100 patients with lung cancer admitted to our hospital from January 2021 to January 2023 were selected as research objects, and were divided into control group and study group, with 50 cases in each group according to different treatment methods. All patients received thoracoscopic surgery, the control group received routine nursing, and the research group received perioperative nursing intervention on the basis of the above, and observed and compared the temperature changes, perioperative complications, postoperative rehabilitation, stress indexes and pain degree of the 2 groups of patients. The temperature at T1, T2, T3, and T4 was lower than that at T0, and the temperature at T3 was the lowest. The temperature at T1, T2, T3, and T4 in the study group was significantly higher than that in the control group (*P* < .05). The incidence of perioperative hypothermia, hypoxemia, chills, and agitation in study group was significantly lower than that in control group (*P* < .05). The anal exhaust time, consciousness recovery time, tracheal extubation time, operating room time, and hospital stay time in the study group were significantly shorter than those in the control group (*P* < .05). After operation, the levels of AD, NA, and Cor in both groups were higher than those before operation, and the levels of AD, NA, and Cor in the study group were significantly lower than those in the control group (*P* < .05). The visual analogue scale scores of the 2 groups at 12 hours and 48 hours after operation were lower than those of the control group at 6 hours, 12 hours, and 48 hours after operation, and the visual analogue scale scores of the study group at 6 hours, 12 hours, and 48 hours after operation were significantly lower than those of the control group (*P* < .05). Nursing intervention during thoracoscopic surgery for lung tumors can stabilize intraoperative and postoperative temperature changes, alleviate stress reaction and pain, reduce the risk of intraoperative hypothermia and hypoxemia, promote the smooth progress of surgery and improve prognosis.

## 1. Introduction

Lung tumors refer to tumors that grow in the lungs, and are mostly divided into benign tumors and malignant tumors. Benign tumors grow slowly, have clear boundaries, and will not spread or metastasize; malignant tumors, also called bronchial lung cancer, have the highest morbidity and mortality rates in the world. primary malignant tumors of the lungs.^[1,2]^ Data show^[3]^ that lung cancer is a malignant tumor that is closely related to bad living habits and endangers people’s lives. It has become the most common cause of death in 28 developed countries around the world, and in at least 35 countries, lung cancer ranks first among men. Cancer is the leading cause of death, second only to breast cancer in female mortality, and has become a global public health problem. In my country, the incidence of lung cancer has been increasing year by year for a relatively long period of time, and it currently ranks fourth among male malignant tumors and fifth among female malignant tumors, increasing the burden on families and socioeconomics.^[4]^ There are no typical characteristics of lung tumors in the early stages. A few patients have symptoms such as chest tightness, coughing, and hoarseness, which are not easy to attract patients’ attention. Later, when infected, there is excessive phlegm and intermittent hemoptysis. When confirmed by imaging and cytology examinations, pathological changes in the lung parenchyma have already appeared. Changes, distant metastasis may occur, seriously endangering their lives.^[5,6]^ Therefore, early detection, diagnosis, and treatment are of great significance to improve the prognosis and quality of life.

Surgery is the preferred way to treat lung tumors. Traditional lobectomy is more invasive, has more postoperative complications, and takes longer to recover. It can no longer meet patients’ needs for clinical services. Segmentectomy mainly removes lesions that contain the disease. The local lung segments of the tissue ensure the structural and functional integrity of other normal tissues in the lung lobes. However, there is no significant anatomical gap between the lung segments. The anatomical operation is complex and difficult, and it is easy to damage adjacent normal lung tissue, making disease treatment more difficult and surgical risk of complications.^[7,8]^ Therefore, finding safe and efficient surgical procedures is an important challenge for cardiopulmonary scientists. As a new minimally invasive thoracic surgery, video-assisted thoracoscopic surgery has the advantages of small trauma, high safety, fast postoperative recovery, and wide range of indications, and has achieved remarkable clinical results.^[9]^ However, due to factors such as complex operations, long operation time, and low temperatures of disinfectant and infusion solutions during thoracic surgery, patients are prone to hypothermia during the operation, and it is difficult to raise body temperature through self-regulation, and it is easy to induce cardiac function. Complications such as coagulation disorders and chills are not conducive to the smooth progress of surgery and postoperative recovery.^[10]^ At present, domestic and foreign research on thoracoscopy for lung tumors mostly focuses on optimizing the surgical path and improving the prognosis. Research on intraoperative body temperature changes is still in the exploratory stage.^[11,12]^ In view of the above problems, this study selected 100 inpatients with lung tumors admitted to our hospital from January 2021 to January 2023 as the research subjects to explore the effects of nursing intervention on temperature changes and perioperative complications in patients with lung tumors who underwent thoracoscopic surgery: symptoms, postoperative recovery, stress indicators, and pain levels, etc, in order to provide scientific basis for improving patients’ perioperative quality of life. The report is as follows.

## 2. Materials and methods

### 2.1. General information

This study selected 100 inpatients with lung tumors admitted to our hospital from January 2021 to January 2023 as the research subjects. This study is a retrospective study, by sorting out the case data, according to different treatment methods divided into control group and study group according to different treatment methods, with 50 cases in each group. The 2 groups of patients were divided into 2 groups according to gender and age. Compared with other general data, the difference is not statistically significant (*P* > .05) and is comparable. All patients underwent thoracoscopic surgery, the control group received routine care, and the research group received perioperative nursing intervention on the basis of the above. This study was reviewed and approved by the Affiliated Hospital of Jiangnan University Medical Ethics Committee (2023-K2022-0-117).

### 2.2. Inclusion and exclusion criteria

Inclusion criteria: ① meet the diagnostic criteria for lung tumors by the American College of Chest Physicians^[13]^; ② diagnosed with lung tumors through CT, chest X-rays, and pathological tissue biopsy, and chest CT images show lung tumors the maximum diameter of the tumor is ≤2 cm; ③ aged between 18 and 80 years old, with normal basal metabolism and body temperature maintained at 36.5 to 37.5 °C; ④ no other lung cancer surgeries and treatments; ⑤ all underwent thoracoscopic radical resection of lung cancer, and the surgical approach was: single hole or multiple holes, operation time <3 hours; ⑥ no cognitive impairment or mental disorder, and can communicate normally; ⑦ research subjects and relatives are informed of the research content and voluntarily sign an informed consent form.

Exclusion criteria: ① patients with severe heart, blood vessel, liver, kidney, and other functional disorders or other malignant tumors; ② patients with secondary tumors or distant metastasis; ③ the minimum value of continuous intraoperative body temperature monitoring data is lower than 36.0 °C; ④ pleural those who have extensive adhesions in the cavity and cannot successfully undergo thoracoscopic surgery or who are converted to thoracotomy during the operation; ⑤ those who have coagulation function, autoimmune system diseases, acute and chronic serious infections; ⑥ those who withdraw from the study midway.

### 2.3. Research methods

All patients underwent thoracoscopic surgery, and the control group received routine care: after admission, patients received routine preoperative health education and visits to understand their condition and psychological state, and were informed of their disease treatment plans, expected treatment effects and nursing plans, so as to promote their confidence in disease treatment; assess anesthesia risks based on the patient’s surgical conditions, vital signs, etc. The operating room temperature should be adjusted to 22 to 25 °C 30 minutes before the patient enters the room. If the temperature needs to be adjusted during the operation, it must be controlled within ±2 °C of the original temperature, the humidity is controlled at 50% to 60%. After instructing the patient to adjust his position, local skin disinfection and draping are carried out, and vital signs are checked. During the operation, a medical heating blanket is used to preheat the mattress and the temperature is maintained at 40 °C. A small quilt is used for the exposed parts. Cover; intraoperative infusion fluids need to be heated to 36 to 38 °C in a preheating box 1 hour before infusion, and the pleural lavage fluid is also heated to 38 to 40 °C. After the operation is completed, the patients are sent to the anesthesia recovery room, where their vital signs and electrocardiogram are monitored, and the room temperature is maintained at 22 to 25 °C.

On the basis of the above, the research team provides perioperative nursing intervention: ① before surgery: conduct in-depth communication with the patient and his family, understand their disease awareness, education level, etc, and introduce to them disease-related knowledge, thoracoscopic surgery procedures, possible adverse reactions and precautions, increase their attention to intraoperative hypothermia, obtain their disease treatment and care compliance, provide them with encouragement and support, relieve their tension and fear, and avoid negative impacts on the operation; inform them before surgery. Quit smoking for 2 weeks to keep the respiratory tract open. For patients with dyspnea, ultrasonic atomization can be used to discharge respiratory secretions, and encourage them to exercise and breathe training to improve cardiopulmonary function. Fasting for 12 hours and drinking for 6 hours before surgery can avoid nausea during the surgery. Vomiting leads to aspiration pneumonia, suffocation, etc. The variable temperature blanket was heated to 40 °C for preheating 1 hour before the operation, and a constant temperature box was used to heat infusion fluids, thoracic flushing fluids, etc, and blood products were maintained at 30 °C, the same as the control group. ② During the operation: after entering the room, monitor the patient’s electrocardiogram, respiration, pulse, body temperature changes, etc, and inhale low-flow and high-concentration oxygen; place a warm heat exchanger above the airway tube to ensure that the patient’s respiratory temperature and humidity remain constant, and reduce the respiratory mucosa. Stimulated by low temperature and long-term ventilation; place the heating blanket on the bed sheets to continuously warm the patient to ensure that the temperature is controlled at 38 to 43 °C. Use rectal temperature monitoring to detect body temperature. If the temperature difference exceeds 2 °C, stop the variable temperature blanket in time and appropriately reduce the blower temperature. During the operation, pay attention to covering the patient with quilts and other warm objects to avoid shifting or slipping, and rub the ends of his limbs to promote distal blood circulation. ③ After the operation: after the operation, the patient is pushed to the anesthesia recovery room, the room temperature is adjusted, and the body position is adjusted to a supine position without pillows to avoid aspiration of secretions, suffocation, etc; the patient’s position, tightness of the restraints, etc are regularly adjusted, and the limbs are massaged regularly to assist turn over and buckle the patient’s back and change positions to avoid deep vein thrombosis in the lower limbs; provide appropriate drug control according to the degree of pain and agitation to prevent or avoid agitation during the awakening period; assist the patient to take deep breaths, cough and expectorate after waking up, and observe his respiratory rate, Rhythm, etc to ensure smooth breathing and avoid dyspnea, cyanosis, etc.

### 2.4. Observation indicators

① Temperature changes: monitor the body temperature at each time period [when entering the room (T0), 20 minutes during the operation (T1), 40 minutes during the operation (T2), 60 minutes during the operation (T3), and after the operation (T4)]. ② The occurrence of perioperative complications: record the occurrence of hypothermia, hypoxemia, shivering, agitation, etc; among them, when the intraoperative core body temperature is <36 °C, it can be judged as hypothermia^[14]^; intraoperative blood oxygen saturation (hypoxemia can be determined when SaO_2_) <90% and oxygen partial pressure <60 mm Hg.^[15]^ ③ Postoperative recovery: record the patient’s postoperative anal exhaust time, consciousness recovery time, tracheal extubation time, time out of the operating room, hospitalization time, etc. ④ Stress indicators: take 3 mL of peripheral venous blood before and after surgery, centrifuge at low speed for 15 minutes at 3000 r/min, let it stand for 10 minutes, and take the supernatant for testing. Enzyme-linked immunosorbent assay was used to detect epinephrine (adrenaline, epinephrine, AD) and norepinephrine (noradrenaline, NA); chemiluminescence method was used to detect serum cortisol (cortisol, Cor) levels, and the kit was provided by Shanghai Enzyme Biotechnology Ltd., Shanghai City operate strictly in accordance with the requirements of the kit. ⑤ Pain level: use the visual analogue scale^[16]^ (VAS) to measure the pain level 6h, 12h, and 48h after surgery, using a 10cm ruler to measure, where 0 means no pain at all; 1 to 3 It is divided into mild pain, no impact; 4 to 6 points, moderate pain, tolerable; 7 to 9 points, severe pain, unbearable but improved after taking analgesics; 10 points, severe pain, unbearable, need to take medication. Useless afterwards.

### 2.5. Statistical processing

SPSS 24.0 statistical software was used. Measurement data that conformed to normal distribution were expressed as ±s, and comparison between groups was performed using *t* test. Enumeration data were expressed as number of cases (n) and percentage (%). Comparison between groups was performed using *χ*^2^ test, with *P* < .05 indicates a statistically significant difference.

## 3. Results

### 3.1. Comparison of basic information

The results showed that there was no statistically significant difference in basic information such as gender, age, BMI, weight, pathological type, tumor type, and anesthesia grade between the 2 groups of patients (*P* > .05). See Table 1.

**Table 1:** Comparison of basic data between the 2 groups.

Group		Control group (n = 50)	Research group (n = 50)	*t*	*P*
Gender (n)	Male	29 (58.00)	28 (56.00)	6.319	.054
	Female	21 (42.00)	22 (44.00)		
Age (year)		42.95 ± 5.37	45.68 ± 5.30	5.084	.901
BMI (kg/m^2^)		23.62 ± 1.06	23.67 ± 1.08	4.797	.505
Weight (kg)		60.36 ± 4.95	60.38 ± 4.91	5.670	.419
Pathological type (n)	Benign tumor	35 (70.00)	34 (68.00)	5.081	.931
	Lung cancer	15 (30.00)	16 (32.00)		
Tumor type	Peripheral type	31 (62.00)	33 (66.00)	5.604	.086
	Central type	19 (38.00)	17 (34.00)		
(n)	I Grade	23 (46.00)	22 (44.00)	4.092	.174
	II Grade	27 (54.00)	28 (56.00)		

### 3.2. Comparison of temperature changes

The results showed that there was no statistically significant difference in the temperature of the 2 groups of patients at T0 (*P* > .05); the temperatures of the 2 groups of patients at T1, T2, T3, and T4 were all lower than those at T0, and the temperatures at T3 were all reduced to the lowest. The research group: the temperatures at T1, T2, T3, and T4 were significantly higher than those in the control group, and the differences were statistically significant (*P* < .05). See Table 2.

**Table 2:** Comparison of temperature changes between the 2 groups ($\bar{x}\pm \;s$, °C).

Time	Control group (n = 50)	Research group (n = 50)	*t*	*P*
T0	36.85 ± 0.29	36.81 ± 0.26	4.958	.082
T1	36.60 ± 0.23	36.74 ± 0.24*	8.623	.005
T2	36.26 ± 0.21	36.68 ± 0.22*	7.064	.001
T3	36.11 ± 0.20	36.40 ± 0.22*	5.942	.002
T4	35.97 ± 0.21	36.64 ± 0.23*	5.879	.006

* Compared with T0, *P* < .05.

### 3.3. Comparison of perioperative complication rates

The results showed that the incidence rates of perioperative hypothermia, hypoxemia, shivering, and agitation in the study group were significantly lower than those in the control group, and the difference was statistically significant (*P* < .05). See Table 3.

**Table 3:** Comparison of the incidence of perioperative complications between the 2 groups (cases, %).

Group	Control group (n = 50)	Research group (n = 50)	*t*	*P*
Hypothermia	4 (8.00)	0 (0.00)	7.623	.000
Hypoxemia	6 (12.00)	1 (2.00)	8.618	.002
Chills	7 (14.00)	1 (2.00)	4.603	.009
Restless	5 (10.00)	1 (2.00)	5.971	.030

### 3.4. Comparison of postoperative recovery conditions

The results showed that the anal exhaust time, consciousness recovery time, tracheal extubation time, operating room time, and hospitalization time in the study group were significantly shorter than those in the control group, and the differences were statistically significant (*P* < .05). See Table 4.

**Table 4:** Comparison of postoperative rehabilitation between the 2 groups ($\bar{x}\pm \;s$).

Time	Control group (n = 50)	Research group (n = 50)	*t*	*P*
Anal exhaust time (h)	44.84 ± 9.36	30.56 ± 8.29	10.694	.006
Consciousness recovery time (h)	12.03 ± 3.27	10.09 ± 2.58	9.680	.001
Tracheal extubation time (h)	16.02 ± 7.51	13.48 ± 5.65	4.036	.000
Time to leave the operating room (h)	81.69 ± 25.36	71.64 ± 20.95	5.697	.005
Length of stay (d)	16.39 ± 4.06	13.28 ± 3.47	5.628	.004

### 3.5. Comparison of stress indicators

The results showed that there was no statistically significant difference in the levels of AD, NA, and Cor between the 2 groups of patients before surgery (*P* > .05); the levels of AD, NA, and Cor in the 2 groups of patients after surgery were all higher than before surgery, and the AD in the study group, NA, and Cor levels were significantly lower than those in the control group, and the differences were statistically significant (*P* < .05). See Table 5.

**Table 5:** Comparison of stress indexes between the 2 groups ($\bar{x}\pm \;s$).

Group	Time	Control group (n = 50)	Research group (n = 50)
AD (pmol/L)	Before surgery	108.62 ± 8.97	105.49 ± 8.95
	After surgery	164.54 ± 14.35*	138.57 ± 10.26*^,^†
NA (μg/L)	Before surgery	234.06 ± 18.67	230.65 ± 18.65
	After surgery	387.95 ± 24.71*	304.52 ± 20.54*^,^†
Cor (μmol/L)	Before surgery	449.65 ± 48.62	448.37 ± 47.95
	After surgery	729.65 ± 75.83*	473.62 ± 53.67*^,^†

* Compared with before surgery, *P* < .05.

† Compared with the control group, *P* < .05.

### 3.6. Comparison of pain levels

The results showed that the VAS scores of the 2 groups of patients at 12 hours and 48 hours after surgery were lower than those at 6 hours after surgery. The VAS scores of the study group at 6 hours, 12 hours, and 48 hours after surgery were significantly lower than those of the control group, and the difference was statistically significant (*P* < .05). See Table 6.

**Table 6:** Comparison of pain degree between the 2 groups ($\bar{x}\pm \;s$, fraction).

Time	Control group (n = 50)	Research group (n = 50)	*t*	*P*
6 hours after surgery	7.62 ± 1.95	6.28 ± 1.69	9.623	.006
12 hours after surgery	6.43 ± 1.61	4.95 ± 1.37*	8.647	.007
48 hours after surgery	2.84 ± 0.97	1.72 ± 0.60*	5.905	.002

* *P* < .05 compared with 6 hours after surgery.

## 4. Discussion

Lung tumors are one of the tumors that originate from bronchial mucosal cells or glands in the lungs. They have the characteristics of high morbidity and mortality. The etiology and pathogenesis of lung tumors are relatively recurrent and are closely related to lifestyle, dietary structure, occupational exposure and genetics. Closely related, long-term smoking, air pollution, etc can also induce the reduction of bronchial glands or lung epithelial cells, leading to lung gene mutations, immune system imbalance, inducing lung tumors, and endangering people’s lives and health.^[17,18]^ Clinical treatment of lung tumors is mostly surgical. VATS can not only remove benign lung tumors, but also lung cancer tissue with a tumor diameter of ≤ 2 cm. It can completely remove the diseased tissue and ensure the function of the lung tissue to the greatest extent. It has It has the advantages of high safety, less trauma, and quick postoperative recovery, which can effectively improve the quality of life and postoperative survival rate of lung tumor patients.^[19]^ Some studies have found^[20]^ that during the actual application of thoracoscopic surgery, due to various factors, the patient’s intraoperative body temperature will be lower than normal, which will increase the risk of intraoperative bleeding, wound infection, etc, affecting the normal operation. In order to improve the safety of surgery, monitoring changes in body temperature during the perioperative period and taking corresponding protective measures are the focus of medical staff in the operating room. This study provides nursing intervention to lung tumor patients undergoing thoracoscopic surgery, which can effectively stabilize perioperative body temperature fluctuations, reduce the incidence of hypothermia and shivering, and control surgical stress reactions and pain levels. It is of great significance to disease treatment and prognosis.

Compared with open surgery, thoracoscopic surgery makes 3 incisions with a length of about 1 to 2 mm on the chest wall, places the endoscope in the patient’s chest, and uses supporting endoscopic instruments and equipment to perform lung tumor resection with minimal damage., mild pain, quick postoperative recovery, high safety, and small postoperative scars, it has become the main method of lung tumor treatment.^[21]^ Some studies have pointed out^[22]^ that the patient’s body temperature level during thoracoscopic surgery shows a dynamic change curve. From a physiological perspective, a constant normal body temperature is the basis for maintaining the body’s metabolism and physiological functions. It maintains a dynamic equilibrium state through the process of heat production and heat dissipation. The decrease in body temperature during surgery indicates that the body’s ability to regulate body temperature decreases and enters a decompensation period. The body’s heat production per unit time is far less than its heat dissipation. Perioperative hypothermia mainly refers to core body temperature within the range of 34 to 36 °C. It has become a common complication in surgical operations, with an incidence rate as high as 50% to 90%. The factors causing hypothermia are complex, including limited conditions, patients during surgery. The subjective temperature is lower, the operation time is longer, large amounts of intraoperative fluids and blood are transfused, and body surface heat is dissipated. In addition, intraoperative anesthetic drugs can also inhibit the temperature regulation center and reduce the contraction function of skeletal muscles. At the same time, the patient’s fear, nervousness, and other emotions will also be aggravated. The degree of imbalance in the thermoregulatory system causes intraoperative hypothermia and hypoxemia.^[23,24]^ Long-term hypothermia will cause the body’s enzymes and liver metabolism to slow down, renal blood flow to decrease, delay the metabolism and excretion rate of anesthetic drugs, prolong postoperative recovery time and tracheal extubation time; it will also reduce platelets and increase blood viscosity increases peripheral vascular resistance, promotes vasoconstriction, increases the risk of tissue ischemia and hypoxia, and affects coagulation function, causing intraoperative bleeding and increasing the risk of bleeding; in addition, low temperature can induce an increase in blood pressure and increase the body’s response to exciting reaction will increase wound infection and arrhythmia symptoms, affecting the overall surgical effect and postoperative recovery effect. Therefore, it is particularly critical to maintain body temperature stability and prevent hypothermia during thoracoscopic surgery.^[25,26]^ Ji J et al^[27]^ implemented perioperative temperature management intervention in patients undergoing selective thoracoscopic lobectomy, which can effectively stabilize the temperature during the operation, reduce the incidence of nausea and vomiting, hypothermia, and shivering, and help improve the quality of postoperative recovery. The data of this study pointed out that the temperature of the 2 groups of patients at T1, T2, T3, and T4 was lower than that at T0, and the temperature at T3 was the lowest. The temperature of the study group at T1, T2, T3, and T4 was significantly higher than that of the control group; The incidence rates of perioperative hypothermia, hypoxemia, shivering, and agitation in the study group were significantly lower than those in the control group. The results are similar to the Ji J literature, indicating that nursing intervention during thoracoscopic surgery can stabilize perioperative temperature changes in patients with lung tumors and reduce the risk of intraoperative and postoperative complications. It may be that informing patients of disease-related knowledge and surgical plans before thoracoscopic surgery can improve patients’ awareness of treatment plans, reduce their anxiety and uneasiness, and improve their compliance with surgical treatment; during the operation, the temperature and humidity of the operating room can be adjusted. Humidity, heated electric blankets, covered with thin quilts and other meals can reduce the loss of skin to the environment during surgery, maintain the core body temperature within a safe range within 1 hour of anesthesia induction, and reduce hypothermia, hypoxemia and circulation abnormalities occur; in addition, constant temperature treatment of infusion fluids, flushing fluids, blood products, etc can reduce the body’s stress response to a certain extent, promote normal blood circulation, reduce heat loss, and reduce the risk of complications.^[28]^

Thoracoscopic surgery is a strong stressor. Various indwelling catheters, surgical operations and anesthetic drug residues will stimulate sympathetic nerve excitement, cause changes in the sympathetic-adrenal medulla and hypothalamic–pituitary–adrenocortical axis, and promote abnormal hormone levels in the body. expression, causing a strong stress response. In addition, due to the patient’s limited knowledge of the disease, interference with clinical symptoms, hypothermia and related complications, the stress response is enhanced, the immune function is disrupted, and the postoperative recovery is affected.^[29]^ AD, NA, and Cor are commonly used indicators to evaluate the degree of stress response of the body. When the body is stimulated by internal and external environments, their levels increase abnormally. Among them, AD and NA have the effect of excitating sympathetic nerves. Excessive secretion can cause increased blood pressure, Abnormal heart rate, etc. Providing health education during thoracoscopic surgery can help patients improve their disease awareness, master surgical methods, anesthesia methods, possible complications, etc, reduce psychological burden, reduce psychological stress reactions, and reduce or prevent postoperative agitation; perioperative period vital signs, temperature changes, etc are closely monitored, and a series of insulation measures are used to keep the body in a relatively stable state, reduce intraoperative and postoperative complications, environmentally friendly postoperative pain, further the body’s stress response, and promote the smooth progress of the operation, which is conducive to Rapid recovery after surgery.^[30]^ The results of this study show that the anal exhaust time, consciousness recovery time, tracheal extubation time, operating room time, and hospitalization time in the study group were all significantly shorter than those in the control group; the AD, NA, and Cor levels of the 2 groups of patients after surgery were higher than those before surgery. The levels of AD, NA, and Cor in the study group were significantly lower than those in the control group; the VAS scores of the 2 groups of patients at 12 hours and 48 hours after surgery were lower than those at 6 hours after surgery, and the VAS scores of the study group at 6 hours, 12 hours, and 48 hours after surgery were all significantly higher. lower than the control group. It is suggested that in patients with lung tumors undergoing thoracoscopic surgery, nursing intervention can reduce the stress response during surgery and postoperative pain, and promote their early recovery.

This study still has limitations, mainly reflected in the small sample size, and lung tumor patients have different disease progression and physical tolerance levels. There may be a certain degree of bias in experimental data and reagent data; while the small sample size has been mentioned, another limitation is that it was conducted as a single-center study. The single-center design may limit the generalizability of our findings to broader, more diverse populations and clinical settings. Variations in institutional practices, patient demographics, and regional factors could influence the observed outcomes. Intraoperative body temperature changes are affected by many factors, lack of systematic insulation measures; the follow-up time was short, and the impact of nursing intervention on the efficacy of thoracoscopy and quality of life was not evaluated. Therefore, further prospective, multicenter research and analysis needs to be designed on more samples that meet the criteria to obtain more data to support the reliability of the research results. Future multi-center studies are recommended to verify the generalizability of the results. These studies could explore the specific effects of different types of nursing interventions on postoperative recovery or employ prospective designs to validate and expand upon our current findings.

In summary, nursing intervention during thoracoscopic surgery can help maintain the intraoperative body temperature of lung tumor patients, reduce the incidence of hypothermia and other complications, reduce stress reactions and postoperative recovery indicators, and promote their early recovery.

## Author contributions

**Conceptualization:** Xiao Qu, Na Hu, Liyan Zhou.

**Data curation:** Xiao Qu, Na Hu, Liyan Zhou.

**Formal analysis:** Xiao Qu, Liyan Zhou.

**Funding acquisition:** Liyan Zhou.

**Investigation:** Xiao Qu, Na Hu.

**Methodology:** Xiao Qu, Na Hu, Liyan Zhou.

**Supervision:** Xiao Qu.

**Validation:** Na Hu.

**Writing – original draft:** Xiao Qu, Liyan Zhou.

**Writing – review & editing:** Xiao Qu, Liyan Zhou.
